# Electrochemical Behavior of Reduced Graphene Oxide Supported Germanium Oxide, Germanium Nitride, and Germanium Phosphide as Lithium-Ion Battery Anodes Obtained from Highly Soluble Germanium Oxide

**DOI:** 10.3390/ijms24076860

**Published:** 2023-04-06

**Authors:** Alexey A. Mikhaylov, Alexander G. Medvedev, Dmitry A. Grishanov, Timur M. Fazliev, Vasilii Chernyshev, Elena A. Mel’nik, Tatiana A. Tripol’skaya, Ovadia Lev, Petr V. Prikhodchenko

**Affiliations:** 1 Kurnakov Institute of General and Inorganic Chemistry, Russian Academy of Sciences, Leninskii prosp. 31, 119991 Moscow, Russia; 2 The Casali Center of Applied Chemistry, The Institute of Chemistry, The Hebrew University of Jerusalem, Jerusalem 9190401, Israel; 3 The Harvey M. Krueger Family Center for Nanoscience and Nanotechnology, The Hebrew University of Jerusalem, Edmond J. Safra Campus, Jerusalem 9190401, Israel

**Keywords:** germanium dioxide, germanium nitride, germanium phosphide, highly soluble germanium oxide, graphene oxide, coating, anode material, lithium-ion battery

## Abstract

Germanium and germanium-based compounds are widely used in microelectronics, optics, solar cells, and sensors. Recently, germanium and its oxides, nitrides, and phosphides have been studied as active electrode materials in lithium- and sodium-ion battery anodes. Herein, the newly introduced highly soluble germanium oxide (HSGO) was used as a versatile precursor for germanium-based functional materials. In the first stage, a germanium-dioxide-reduced graphene oxide (rGO) composite was obtained by complete precipitation of GeO_2_ nanoparticles on the GO from an aqueous solution of HSGO and subsequent thermal treatment in argon at low temperature. The composition of the composite, GeO_2_-rGO (20 to 80 wt.% of crystalline phase), was able to be accurately determined by the HSGO to GO ratio in the initial solution since complete deposition and precipitation were achieved. The chemical activity of germanium dioxide nanoparticles deposited on reduced graphene oxide was shown by conversion to rGO-supported germanium nitride and phosphide phases. The GeP-rGO and Ge_3_N_4_-rGO composites with different morphologies were prepared in this study for the first time. As a test case, composite materials with different loadings of GeO_2_, GeP, and Ge_3_N_4_ were evaluated as lithium-ion battery anodes. Reversible conversion–alloying was demonstrated in all cases, and for the low-germanium loading range (20 wt.%), almost theoretical charge capacity based on the germanium content was attained at 100 mA g^−1^ (i.e., 2595 vs. 2465 mAh g^−1^ for Ge_3_N_4_ and 1790 vs. 1850 mAh g^−1^ for GeP). The germanium oxide was less efficiently exploited due to its lower conversion reversibility.

## 1. Introduction

Germanium and binary inorganic germanium compounds (GeO_2_, GeP, Ge_3_N_4_) are widely used in various fields of science and technology [1]. Germanium is a critically important semiconductor for microelectronics, optics, solar cells, and sensing. The applications of germanium dioxide are determined by its optical and electrical properties [2] and are characterized by a wide band gap (more than 5 eV), high transparency in the visible and infrared regions, and a high refractive index (1.6–1.7) [3,4]. Germanium phosphide has emerged as an attractive candidate for broad-band and mid-infrared photonics [5]. Germanium nitride has attracted interest due to its unique properties [6,7,8]. It can be used as a thin film material for the passivation of semiconductors [9,10], for plasmonic devices [1], and as an effective non-oxide photocatalyst for water splitting [11]. Germanium has higher electrical and ionic conductivity than silicon, which makes it an attractive alternative for high-performance anode material [12,13,14]. Despite its high cost, elemental germanium and its compounds are promising electrode materials in metal-ion batteries [15,16,17,18] due to the large theoretical gravimetric and volumetric specific capacities of germanium (with the formation of Li_15_Ge_4_), equal to 1384 mAh g^−1^ and 7366 mAh cm^−3^, respectively. Unfortunately, large volume changes (by more than 300%) during Li alloying/de-alloying lead to the pulverization of electrode particles and destabilization of solid electrolyte interphase (SEI) films, which hinders the use of germanium-based materials in LIBs [19,20]. Various approaches have been proposed to prevent a dramatic capacitance decrease in Ge-based anodes. For example, the synthesis of carbon-containing composites [21,22,23,24], which endow elasticity and conductive network, can significantly increase the long-cycling stability of the anode material. In addition, fabricating nanosized particles or particles of a tailored morphology [20,25,26] is an effective strategy to avoid degradation during cycling. Nowadays, different germanium compounds and their composites are studied as active materials for electrodes of lithium-ion batteries: GeO_2_ [27,28,29], Ge_3_N_4_, GeP of different stoichiometry [30,31,32,33,34,35,36], and germanium chalcogenides (sulfides, selenides, tellurides) [37,38,39].

Germanium phosphide, which combines the benefits of both germanium and phosphorus, is considered a potential anode for lithium- and sodium-ion batteries. Currently, the main approaches to synthesizing nanosized germanium phosphide are limited to ball milling and separation of GeP particles. The ball milling leads to the formation of irregularly shaped particles with a large size spread (from several tens of nanometers to several microns) [40]. Meanwhile, exfoliated GeP showed relatively low capacity and cyclic stability [35]. Microspheres of germanium phosphide prepared by a solvothermal method showed attractive electrochemical properties as a potential anode material, such as a high first-cycle Coulombic efficiency of 83% and high reversible capacity (1400 mAh g^−1^ after 150 cycles at 0.2 C) [41]. Germanium phosphide particles were obtained by red phosphorus evaporation–condensation on the germanium substrate. The authors demonstrated that the morphology was determined by the morphology of the initial germanium reactant [42].

Compared to the phosphide and oxide, germanium nitride has been less studied as an LIB anode material, perhaps since the first publication showed that only 40% of the material participates in the reaction with lithium ions, and unreacted Ge_3_N_4_ remains in the core. Therefore, it was concluded that the conversion reaction forming Li_3_N is irreversible [43].

Germanium nitride is usually obtained by nitridation with gaseous ammonia at high temperatures and a long annealing time [11,44]. Kim et al. described a new technique for obtaining the Ge_3_N_4_@C composite by partial oxidation of germanium and nitridation by gaseous NH_3_ at 700 °C for 1 h. Subsequent carbonization with acetylene at 800 °C results in Ge_3_N_4_@C powder with a specific capacity of 600–700 mAh g^−1^ at 550 mA g^−1^ for 300 cycles as a LIB anode [45].

The introduction of carbon components, such as graphene or carbon nanotubes, can enhance the performance of the germanium oxide LIB anode. The observed reversible capacity was close to or even higher than the theoretical reversible capacity of germanium oxide [23,26,29,46,47,48,49,50]. These observations show the possible partial reversibility of Li_2_O formation. Lv et al. calculated that 44.4–44.6% of Li_2_O matrix participates in the reverse alloying during cycling at 100 mA g^−1^ due to intimate contact between graphene layers and GeO_x_ nanoparticles [51]. However, the significant irreversible capacity loss of the first cycle remains a severe problem for germanium oxide anode materials [52].

We have previously reported on the green-hydrogen-peroxide-assisted sol–gel processing route to deposit thin films of p-block peroxo compounds on various substrates [53,54]. The thermal or chemical treatment of these coatings on graphene oxide layers results in the formation of the corresponding oxides, sulfides, and tellurides, which are effective as lithium-, sodium-, and potassium-ion battery anodes [50,55,56,57,58,59,60,61,62,63,64,65]. In particular, a peroxogermanate thin film was deposited in high yield at room temperature on graphene oxide from peroxogermanate sols [50]. Thermal treatment of the filtered material produced amorphous germanium dioxide, crystalline germanium dioxide, and elemental germanium films supported by reduced graphene oxide (rGO). The obtained composites exhibited high cycling stability and good rate performance as LIB anodes.

The germanium compounds are expensive reagents, which calls for processing protocols with a quantitative yield of germanium. For this, there is a need for new convenient starting germanium compounds with higher reactivity and better water solubility compared to known precursors. Herein, we report an environmentally friendly method for the preparation of germanium-based composite materials from a highly soluble germanium oxide (HSGO) and the performance of the Ge composite products as anode materials in lithium-ion batteries. Germanium phosphide and nitride were selected as target materials for two reasons: (i) The high lithium conductivities of lithium nitride and phosphide—the lithiation by-products of GeP and Ge_3_N_4_. Lithium nitride and lithium phosphide are known lithium superconductors [66,67], and amorphous Li_3_N [68,69] and Li_3_P [67,70] were also reported to exhibit high ionic conductivity (10^−3^ S·cm^−1^). (ii) To improve the performance of germanium compounds as LIB anodes, it is, of course, essential to exhibit high Ge-Li alloying performance, but the performance can be further enhanced by exploiting the charge capacity of the conversion reaction associated with the counter element. Due to the lower electronegativity of nitrogen and phosphorous compared to oxygen, the respective bond cleavage and reversibility of the reactions of lithium phosphide and nitride should be better than Li_2_O.

## 2. Results and Discussion

### 2.1. Synthesis

**Figure 1:**
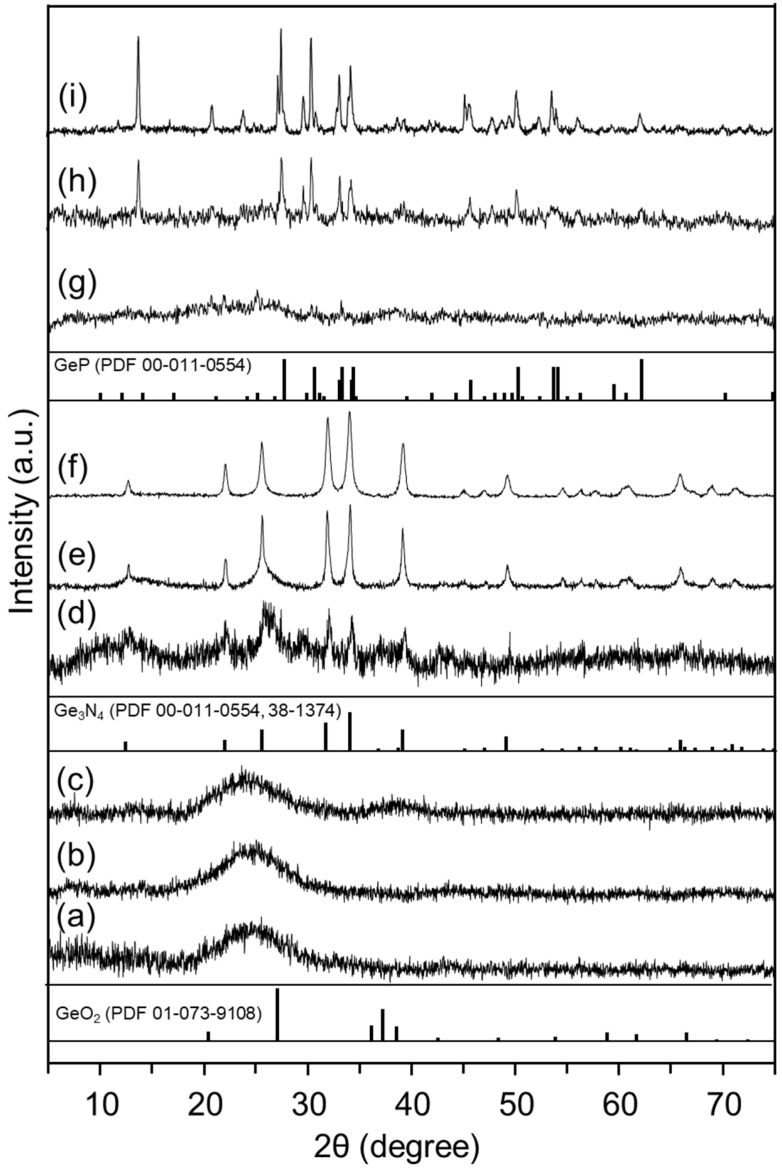
Powder X-ray diffractograms of GeO_2_-rGO-20 (**a**), GeO_2_-rGO-50 (**b**), GeO_2_-rGO-80 (**c**), Ge_3_N_4_-rGO-20 (**d**), Ge_3_N_4_-rGO-50 (**e**), Ge_3_N_4_-rGO-80 (**f**), GeP-rGO-20 (**g**), GeP-rGO-50 (**h**), and GeP-rGO-80 (**i**) powders. Vertical bars below represent the standard diffraction data for corresponding crystalline germanium compounds.

**Figure 2:**
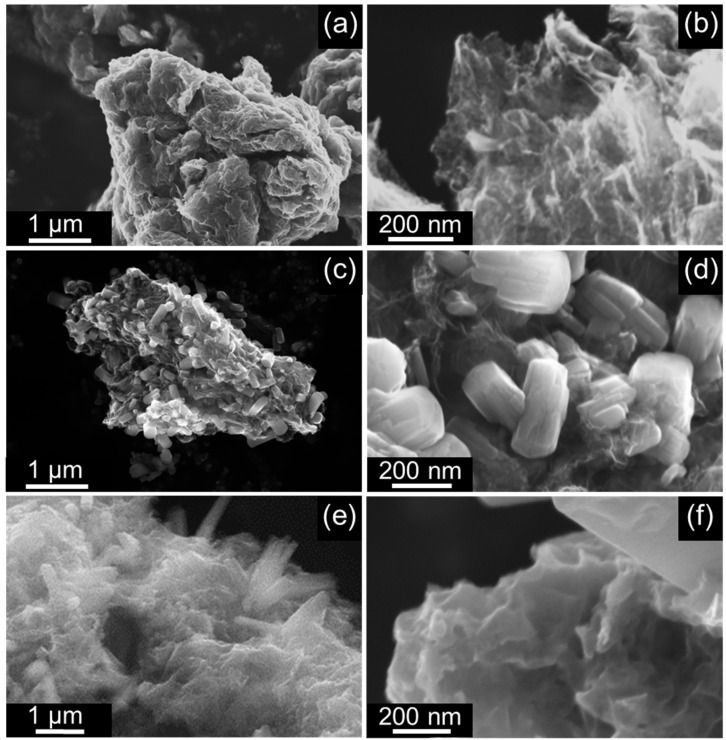
SEM images of GeO_2_-rGO-80 (**a**,**b**), Ge_3_N_4_-rGO-80 (**c**,**d**), and GeP-rGO-80 (**e**,**f**).

The choice of an effective germanium precursor in the form of a highly soluble form of amorphous germanium oxide made it possible accurately dose the amount of germanium introduced into the system and opened new possibilities for controlling the morphology and composition of composite materials. HSGO was obtained by rapid decomposition of ammonium peroxogermanate (NH_4_)_6_[Ge_6_(μ-OO)_6_(μ-O)_6_(OH)_6_].6H_2_O at 300 °C [71]. We have already demonstrated that HSGO has a high solubility (up to 100 g per liter of water) and reactivity and, therefore, is a convenient starting reagent for the preparation of various germanium compounds [71,72]. However, until now, HSGO has not been used to obtain germanium-based composite functional materials or electrode materials. The first stage of synthesis was the deposition of a film of germanium oxide nanoparticles on the surface of graphene oxide sheets from an aqueous solution of HSGO. High solubility of precursors in water allows for easy regulation of the loading of germanium dioxide in obtained materials. Three different loadings of germanium dioxide were chosen for subsequent thermal and chemical processing, namely, 20, 50, and 80 wt.% of GeO_2_. The addition of aqueous ammonia to the solution of HSGO increased the pH from 5.5 to about 8. Precipitation of germanium oxide species on the graphene oxide was then accomplished by the addition of ethanol, resulting in uniform amorphous germanium-based coating (Figure S1). According to X-ray powder diffractometry, the crystalline phase of germanium dioxide was not formed even for the highest loading of germanium (Figure 1a). Subsequent heat treatment at a relatively low temperature of 300 °C for 3 h resulted in the reduction of graphene oxide and the crystallization of nanosized germanium dioxide. The XRD of the product revealed broad low-intensity peaks that were assigned to the GeO_2_ phase (PDF 01-073-9108) (Figure 1c). After the heat treatment, the coating morphology did not contain large agglomerates on the rGO surface and retained the morphology of the initial material (Figure 2a,b and Figure S1).

According to the CHN analysis, the amount of carbon in GeO_2_-rGO composites corresponded to the initial ratio of HSGO to GO, resulting in 16.7, 42.9, and 75.5 wt.% for 20, 50, and 80 wt.% of GO, respectively. The reduction of graphene oxide, accompanied by the removal of oxygen-containing functional groups, decreased the carbon content in the heated materials, GeO_2_-rGO (Table 1). The CHN confirms the high yield of composite material in terms of germanium. Thus, the described procedure was a simple, low-cost, and highly effective method for the preparation of GeO_2_-rGO composite.

The GeO_2_-rGO composites are used as a suitable initial material for preparing crystalline germanium nitride and phosphide-decorated rGO. As far as we know, this is the first attempt to obtain rGO-supported Ge_3_N_4_ and GeP composites. Previously, only the corresponding carbonized phases were obtained and characterized [34,45]. The lack of research can be attributed to the limited number of wet methods for obtaining germanium oxide–graphene oxide composites due to the low solubility of germanium oxide.

Prolonged treatment of GeO_2_-rGO-80 by gaseous NH_3_ at 700 °C for 2 h yields long wires of germanium nitride (Figure S2). Reduced treatment time (up to 5–10 min) results in Ge_3_N_4_ agglomerates with an average size of 50–200 nm, which decorate the rGO surface (Figure 2c,d). It should be noted that a part of the crystalline phase appears as a uniform coating on the surface of reduced graphene oxide (Figure S3). An attempt to control the particle size of germanium nitride was made by adjusting the ratio between the loading of germanium dioxide and graphene oxide. Decreasing of GeO_2_ loading to 20 wt.% (GeO_2_-rGO-20) resulted in a uniform coating of Ge_3_N_4_ crystalline phase, as shown by the SEM and EDX analyses (Figure S4). Treatment of GeO_2_-rGO-50 by ammonia formed a Ge_3_N_4_-rGO-50 composite consisting of rGO decorated with about 100 nm nitride particles. EDX mapping (Figure S5) showed that a thin layer of the Ge_3_N_4_ phase uniformly coated the rGO surface. We were able to estimate the mass content of the germanium nitride phase on the basis of the CHN analysis. The calculated loadings of germanium nitride were 58.7, 45.7, and 33.5 wt.% for Ge_3_N_4_-rGO-80, Ge_3_N_4_-rGO-50, and Ge_3_N_4_-rGO-20, respectively (Table 1). The calculated values indicated the presence of a residual germanium dioxide phase in the Ge_3_N_4_-rGO-80 sample. The powder diffractograms of Ge_3_N_4_-rGO samples reflected the structure of *β*-Ge_3_N_4_ (PDF 00-011-0554, Figure 1d–f). Both samples contained an admixture of a low-crystallinity phase of germanium nitride that appeared as a background and broad peaks according to the corresponding diffractograms. Thus, the Ge_3_N_4_-rGO-50 and Ge_3_N_4_-rGO-80 were refined (by the methodology in reference [73] as composites with bimodal crystallite size distributions. The average crystallite size for the germanium nitride phase, which appeared as a uniform coating, was around 2 nm in all three samples, while the anchored particles were characterized by 20.5 and 31.5 nm in Ge_3_N_4_-rGO-50 and Ge_3_N_4_-rGO-80, respectively. The large particle parameters were able to be significantly affected by crystallite shape.

Heat treatment of GeO_2_-rGO material with red phosphorus in a sealed quartz tube led to the formation of a germanium phosphide phase, which was clearly confirmed by X-ray powder diffraction data (Figure 1g–i). The scanning electron microscopy and energy-dispersive X-ray spectroscopy demonstrated the formation of both a uniform coating of germanium phosphide on the surface of reduced graphene oxide and relatively large particles of germanium phosphide with an average crystallite size of 168 nm (computed by the Scherrer equation), as in the case of germanium nitride with a high loading of germanium dioxide in the starting material (Figure 2e,f and Figure S6). Similar to the synthetic procedure of germanium nitride, the decrease in the content of GeO_2_ to 50% formed particles with a smaller crystallite size of 38.4 nm. Subsequent decreasing of GeO_2_ loading to 20% revealed a uniform coating of reduced graphene oxide by germanium phosphide with an average crystallite size of 2.2 nm (Figures S7 and S8), similar to values obtained by Rietveld refinement of experimental data for materials with higher Ge loading. According to the EDX analysis, the Ge/P ratio corresponded to the GeP crystalline phase (Table 1).

Using HSGO as a precursor allowed us to, firstly, accurately control the amount of active material loaded into the system and, secondly, to form a uniform coating of germanium oxide on graphene oxide. In addition, regulating the germanium oxide-graphene oxide ratio and the reaction time allowed for the obtaining of materials of different morphologies. Thus, highly soluble germanium oxide is a suitable and effective precursor for fabricating germanium-based materials of various compositions and morphologies, which was demonstrated by synthesizing germanium nitride and phosphide composites of reduced graphene oxide. As a test case, all nine obtained composites were evaluated as active materials of anodes in lithium-ion batteries to demonstrate the usefulness, uniqueness, and versatility of the HSGO-based synthesis approach.

### 2.2. Electrochemical Evaluation

**Figure 3:**
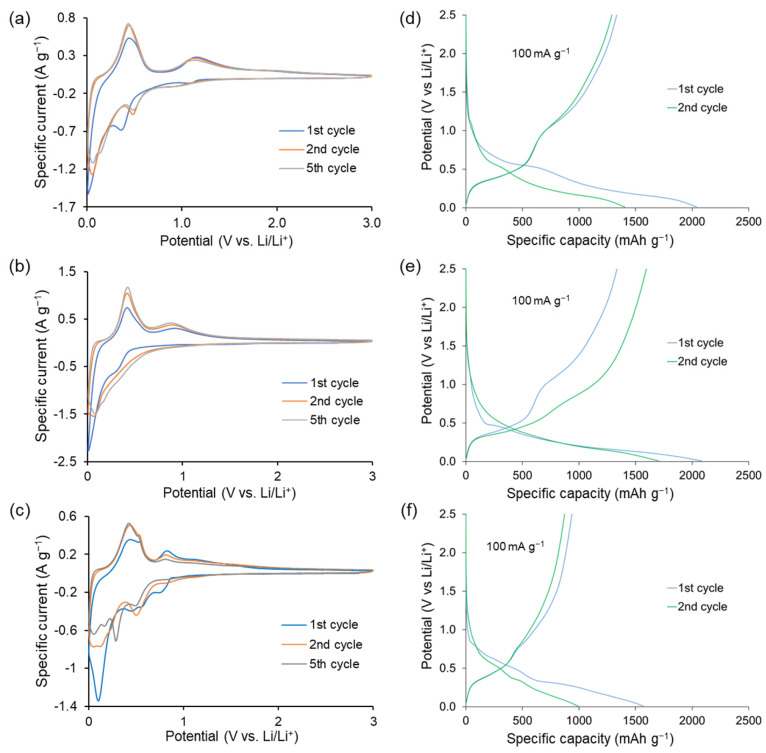
Cycling voltammetry of GeO_2_-rGO-80 (**a**), Ge_3_N_4_-rGO-80 (**b**), and GeP-rGO-80 (**c**) at 0.1 mV s^−1^. First and second charge−discharge curves for GeO_2_-rGO-80 (**d**), Ge_3_N_4_-rGO-80 (**e**), and GeP-rGO-80 (**f**) at 100 mA g^−1^.

Cyclic voltammetry was carried out to determine the electrochemical properties of the composites at room temperature. The CV plot of GeO_2_-rGO-80 is shown in Figure 3a. The first reduction scan shows a shallow shoulder at 1.07, which was previously attributed to minor lithiation of the germanium oxide to form Li_y_GeO_2_. An intensive peak at 0.4 V was attributed to the formation of the SEI layer overlapping a conversion reaction (Li_y_GeO_2_ to Ge(0)). Then, a cathodic wave, at E < 0.2 V, was attributed to a Ge-Li alloying process: (xLi + Ge → Li_x_Ge (0 ≤ x ≤ 4.4)). Similarly, the discharge peaks on subsequent cycles at about 0.98 V and 0.46 V vs. Li/Li^+^ and the cathodic wave at <0.2 V were attributed to minute alloying to form Li_y_GeO_2_, a conversion reaction that produced elemental Ge and then Ge-Li alloying [24,50]. The charging peaks were present at 0.44 and 1.14 V. The first related to dealloying, and the second indicated the (re)oxidation of Ge to GeO_2_ [50,51,74].

The CV plot of the Ge_3_N_4_-rGO-80 anode was similar to the GeO_2_-rGO electrode (Figure 3b) and reflected the alloying–conversion reactions. As indicated by Pereira et al. [43], there is practically only one reduction wave corresponding to the conversion and alloying reaction (Equation (1)).

Ge_3_N_4_ + 3(4 + x)Li^+^ + 3(4 + x)e^−^ → 3Li_x_Ge + 4Li_3_N, (1)

where 0≤ x ≤ 4.4. Pereira et al. [43] attributed the overlap of the alloying and conversion waves to the rapid reductive alloying of Ge(0) to form Li_x_Ge alloy phases. The small shoulder, at 0.2 V, on the first discharging scan may also be attributed to this alloying reaction. The two anodic peaks at 0.4 and 0.9 V were attributed to the delithiation reaction (Li_x_Ge → Ge + xLi^+^ + xe^−^) and the partial recovery of lithium germanium nitride Li_z_Ge_y_N, although full recovery of the original germanium nitride Ge_3_N_4_ was unable to be proven by X-ray, and Pereira et al. [43] did not identify this phase. The persistence of the 0.9 V anodic peak (Figure 3b) showed that the conversion reaction was partially reversible, and nitride (re)formation contributed significantly to the observed charge capacity. The same can be concluded on the basis of the relatively moderate (around 18%) capacity loss in the first cycle. Irreversible formation of the inactive Li_3_N phase was noted [45], and Periera et al. [43] even reported that the 0.9 V peak disappeared after long cycling. Therefore, we carried out cyclic voltammetry of the Ge_3_N_4_-rGO-80 electrode after 150 discharge–charge cycles at 100 mA g^−1^ (Figure S10). The figure clearly indicates the presence of the 0.9 V peak and confirms the capacity contribution of reversible Li_3_N formation even after long cycling. The intimate contact between the germanium nitride coating and graphene oxide could be responsible for the favorable reversibility of the germanium nitride conversion reaction in this study. The CV plots of GeO_2_-rGO-20 and Ge_3_N_4_-rGO-20 are presented in Figure S9. They indicate the validity of our conclusions regarding the lower potential at which the conversion of germanium nitride took place compared to germanium oxide and the higher contribution of the conversion reaction in the former.

Figure 3c depicts the CV plots of the germanium phosphide anode, GeP-rGO-80. The study was conducted under the same conditions used to investigate the germanium oxide and germanium nitride electrodes. Comparison of the CV plots with the literature allows us to assign the following processes [30,32,35,40]. The interval 1.0–0.68 V corresponded to lithium intercalation with the formation of the Li_x_GeP phase. The subsequent stage of conversion with the formation of Li_3_P and Ge appeared as peaks in the range of 0.68–0.40 V. The 0.4–0.005 V peaks were assigned to the alloying stage with Li_y_Ge formation. At the charging scan, the active electrode demonstrated electrochemical oxidation peaks at the 0.005–0.68 V interval assigned to the Li_y_Ge dealloying process. The next potential window at 0.68–1.1 V was attributed to the reversal of the conversion reaction with Li_x_GeP formation. The broad, low-intensity peak at higher potentials was assigned to the deintercalation reaction, which led to the GeP formation. The lower germanium phosphide loading (GeP-rGO-20) provided a similar CV plot (Figure S9c) but with much lower currents and less distinct peaks. The germanium phosphide anodes, similar to the germanium nitride electrodes, exhibited significant reversibility and charge contribution to the conversion reaction. However, in all cases, the alloying reactions contributed a much larger charge capacity than the conversion reactions.

The galvanostatic charge–discharge curves of the three different germanium compounds are presented in Figure 3d–f. The first and second cycles at 100 mA g^−1^ showed good first cycle efficiency of about 68, 80, and 63% for GeO_2_-rGO-80, Ge_3_N_4_-rGO-80, and GeP-rGO-80, respectively, which were not inferior and mostly exceeded the best-published values, including those tested at a narrower potential window of up to 1.5 V [33,43,45,50].

**Figure 4:**
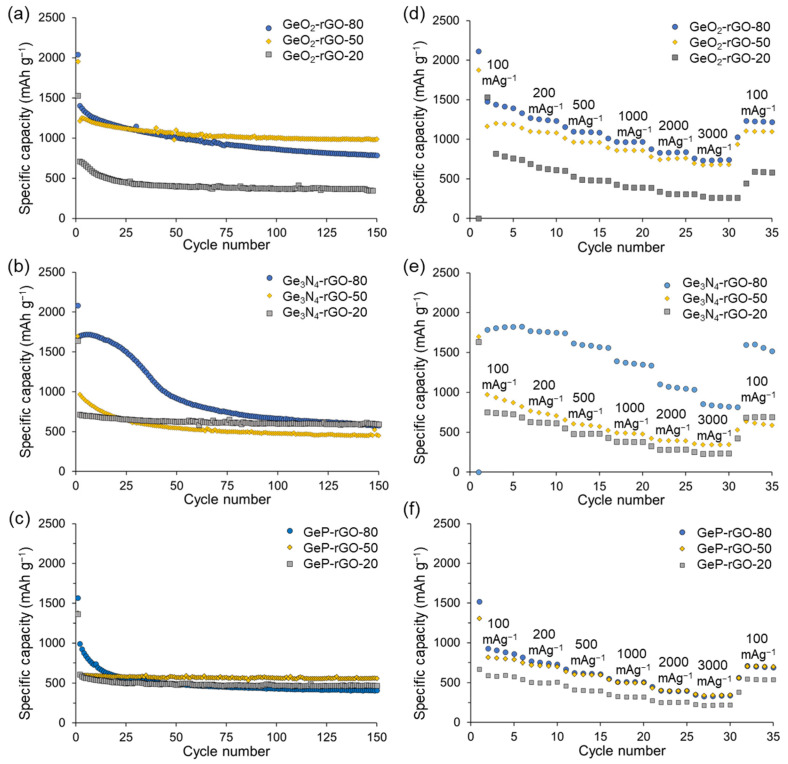
Cycling voltammetry of GeO_2_-rGO-80 (**a**), Ge_3_N_4_-rGO-80 (**b**), and GeP-rGO-80 (**c**) at 0.1 mV s^−1^. First and second charge−discharge curves for GeO_2_-rGO-80 (**d**), Ge_3_N_4_-rGO-80 (**e**), and GeP-rGO-80 (**f**) at 100 mA g^−1^.

The specific capacities depended on the Ge content in the active phase loadings (Figure 4). We carried out long cycling stability tests for 150 cycles at 100 mA g^−1^ and evaluated the rate performance at 100–3000 mA g^−1^ at a potential window of 0.005–3.0 V vs. Li/Li^+^. The decreases in specific capacity after 150 charge–discharge cycles compared to the second cycle were 44% (from 1404 to 785 mAh g^−1^), 66% (from 1696 to 575 mAh g^−1^), and 58% (from 990 to 408 mAh g^−1^) for the GeO_2_-rGO-80, Ge_3_N_4_-rGO-80, and GeP-rGO-80 anodes, respectively. The obtained results were not inferior to the recently reported data for GeO_2_ [23,24,50], Ge_3_N_4_ [43,45], and GeP anodes [35]. This can be attributed to the large volume expansion of the inorganic phase.

The electrode materials with lower germanium content demonstrated good cycling stability for germanium oxide, nitride, and phosphide. The specific cycling capacities of the Ge_3_N_4_-rGO-20 and GeP-rGO-20 were higher than for GeO_2_-rGO-80 after long cycling at 100 mA g^−1^. The capacity drops after 150 cycles were only 17% (from 714 to 592 mA g^−1^) and 23% (from 610 to 468 mA g^−1^) for Ge_3_N_4_-rGO-20 and GeP-rGO-20, respectively, while for GeO_2_-rGO-20 material, it was 51% (from 710 to 345 mA g^−1^). The increased stability at low loading can be attributed to the higher physical buffering of the larger fraction of the (flexible) rGO or to the better adhesion of the small nanoparticles to the rGO surface, having a larger specific contact area with the rGO support (i.e., contact area to the rGO per crystallite weight). Support for the second explanation was indirectly provided by the performance of the GeO_2_ electrodes, which exhibited monomodal particle size distribution (even for the 80 wt.% GeO_2_ loading) but exhibited similar cyclic stabilities at different germanium loadings.

According to CHN analysis, the carbon content in Ge_3_N_4_-rGO-20 and GeP-rGO-20 samples were equal to 76.2 and 74.2 wt.%, respectively (Table 1), corresponding to the ≈24 and ≈26 wt.% content of germanium nitride and phosphide, respectively. Thus, the approximate specific capacity values per gram of Ge_3_N_4_ and GeP were equal to 2595 and 1790 mAh g^−1^ at a 100 mAg^−1^ scan rate, respectively. The obtained values were close to the theoretical capacity for Ge_3_N_4_ (2465 mAh g^−1^) and GeP (1850 mAh g^−1^) charge capacities, indicating highly efficient Ge_3_N_4_-rGO-20 and GeP-rGO-20 electrode materials and the reversibility of Li_3_N matrix formation. In this argument, we ignored the contribution of the graphene oxide to the capacity, which, at most, would amount to 280 mA per g of active germanium compound. Comparison of a CV plot for a germanium nitride anode with higher and lower loadings of active phase (Figure 3b and Figure S9b, respectively) with those for germanium oxide allowed us to conclude with regards to the reversible Li_3_N formation resulting in higher specific capacity of germanium nitride electrodes.

The rate capabilities of germanium dioxide, nitride, and phosphide composite anodes with different loading of reduced graphene oxide at 100 to 3000 mA g^−1^ currents are presented in Figure 4d–f, respectively. All materials demonstrated good high-rate performance. An increase in charge–discharge current by a factor of 30 to 3000 mA g^−1^ resulted in the preservation of 33, 36, and 46% of the specific charge capacity of Ge_3_N_4_-rGO-20, Ge_3_N_4_-rGO-50, and Ge_3_N_4_-rGO-80 materials, respectively (Figure 4e). The GeP-based materials retained around 40% of the initial capacity at a high rate of 3000 mA g^−1^ (Figure 4f). Similar values, equal to 32, 50, and 48%, were obtained for initial germanium dioxide–rGO composites with 20, 50, and 80% loading of the inorganic phase, respectively. To the best of our knowledge, the obtained specific capacity value of 830 mAh g^−1^ at 3000 mA g^−1^ for Ge_3_N_4_-rGO-80 is the highest attained for electrodes based on germanium nitride, while the experimental values for germanium oxide and phosphide are similar to the best-published values [35].

We tested the GeO_2_-rGO-80, Ge_3_N_4_-rGO-80, and GeP-rGO-80 materials for cycling stability at a high rate of 1000 mA g^−1^ (Figure S11). The specific capacity values were equal to 360, 390, and 300 mAh g^−1^ after 500 cycles for GeO_2_-rGO-80, Ge_3_N_4_-rGO-80, and GeP-rGO-80, respectively.

Conversion anodes are characterized by high-voltage hysteresis and, thus, poorer energy efficiency compared to alloying and conversion-alloying anodes [75,76,77,78,79]. Since the electrodes exhibited considerable conversion reaction contribution, the voltage hysteresis was computed. The calculation protocol is provided in the Supporting Information. The average voltage hystereses calculated at the 30th cycle at 100 mA g^−1^ were equal to 0.70, 0.46, and 0.51 V for GeO_2_-rGO-80, Ge_3_N_4_-rGO-80, and GeP-rGO-80, respectively. The germanium nitride and phosphide anodes exhibited lower voltage hystereses compared to the GeO_2_-rGO anode and to other conversion electrodes, and their voltage hystereses were similar to tin sulfide electrodes, which exhibited a voltage hysteresis of 0.48 V [79]. The comparison of the voltage hysteresis, $\bar{\Delta V}$ of several conversion LIB anodes, was reported by Zhong et al. [80]. The best voltage hystereses of LIB anodes, namely, Fe_3_O_4_ and MnO, showed ($\bar{\Delta V}$) > 0.55 V, which was higher than the values obtained for the germanium nitride LIB anode. This was not surprising. The Ge-based anodes studied in this research were better classified as conversion–alloying anodes rather than conversion reaction electrodes.

## 3. Materials and Methods

### 3.1. Synthetic Procedures

#### 3.1.1. Synthesis of Highly Soluble Germanium Oxide (HSGO)

Synthesis was conducted according to [71]. Briefly, 0.5208 g of GeCl_4_ (2.43 mmol) was added to 20 mL of 5 wt.% H_2_O_2_. The precipitate was dispersed in an ultrasonic bath for 2 min. The dispersion was neutralized with 2 mL of NH_4_OH (12.5 wt.% solution) until pH 9. After an additional 5 min of stirring, 20 mL of alcohol was added for full precipitation. The precipitate was separated by filtration on a glass filter and washed three times with 3 mL of 50 wt.% EtOH, once with 3 mL EtOH, and once with 3 mL diethyl ether. A total of 0.3892 g of APG was obtained after drying (92.2% yield based on Ge). Then, 0.389 g of APG was heated in a furnace at 300 °C for 15 min to produce 0.231 g of HSGO powder (98.7% yield based on Ge).

#### 3.1.2. Preparation of Graphene Oxide (GO) Dispersion

GO was synthesized from exfoliated graphite by a modified Hummers method, and a detailed protocol is described in our previous articles [54,58,64].

#### 3.1.3. Synthesis of GeO_2_-rGO

A total of 7.5 g of aqueous GO dispersion (1.2 wt.%) was mixed with 17.5 mL of DIW with sonication. Then, 350 mg of HSGO and 1 mL of NH_4_OH 25 wt.% aqueous solution were introduced. The precipitation of GeO_2_ onto the GO surface was accomplished by the addition of 100 mL of ethanol under vigorous stirring. The coated GO was centrifuged and washed two times with ethanol and once with diethyl ether. The material was dried under vacuum at room temperature and then heated in Ar at 300 °C for 3 h. Samples with different GeO_2_ loading were prepared and assigned as GeO_2_-rGO-80, GeO_2_-rGO-50, and GeO_2_-rGO-20.

#### 3.1.4. Synthesis of Ge_3_N_4_-rGO

GeO_2_-rGO powders with different Ge loading were introduced to the hot tube furnace under dry NH_3_ flow at 700 °C for 10 min. Dark powder was obtained and assigned as Ge_3_N_4_-rGO-80, Ge_3_N_4_-rGO-50, and Ge_3_N_4_-rGO-20.

#### 3.1.5. Synthesis of GeP-rGO

The GeO_2_-rGO materials were mixed with red phosphorous and sealed in a quartz tube. The tube was heated at 590 °C for 2 h. Dark gray powder was collected and assigned as GeP-rGO-80, GeP-rGO-50, and GeP-rGO-20 depending on the initial GeO_2_ loading.

### 3.2. Material Characterization

Details of the material characterization techniques are provided in the Supplementary Information.

### 3.3. Electrochemical Measurements

The active electrode material was mixed with Super P (Alfa Aesar) and polyvinylidene difluoride (PVDF) (Sigma-Aldrich) in a weight ratio of 70:15:15 with N-methyl pyrrolidone (NMP) as the medium to form a slurry. The slurry was then coated on a roughened copper foil as a current collector using a doctor blade. The electrode was then dried at 80 °C and pressed in a roll press. The electrodes were cut into 16 mm diameter discs and further dried at 110 °C for 4 h in a vacuum before being introduced into an argon-filled glovebox. The average loading of active material on the Cu foil was 1.5–2 mg cm^−2^. The electrodes were assembled with Li metal counter electrodes in a 2016-type coin cell. A total of 1 M LiPF_6_ in EC:DEC was used as an electrolyte. Then, 5% *v*/*v* fluorinated ethylene carbonate (FEC, Alfa Aesar) was added to the electrolyte to improve solid electrolyte interface (SEI) stability. The coin cells were assembled in an Ar-filled glovebox with less than 0.1 ppm oxygen. The charge/discharge tests were performed with a Neware battery tester within a voltage window of 0.01–3.0 V vs. Li/Li^+^ at 100–3000 mA g^−1^ rate. Cyclic voltammetry (0.005–3.0 V, 0.1 mV s^−1^) was performed with a VersaSTAT3 potentiostat.

## 4. Conclusions

As far as we know, this is the first report on the Ge_3_N_4_-rGO and GeP-rGO composites. The highly soluble germanium oxide (HSGO) was used as a versatile precursor of germanium-based electrode materials. HSGO was prepared in mild conditions with high yield from the germanium coordination peroxo compound, namely, ammonium peroxogermanate (APG), (NH_4_)_6_[Ge_6_(μ-OO)_6_(μ-O)_6_(OH)_6_]·6H_2_O. In the first stage, HSGO was used as a precursor for the deposition of uniform coating consisting of germanium dioxide nanoparticles on reduced graphene oxide (GeO_2_-rGO). The loading of GeO_2_ phase was regulated by the ratio of HSGO and graphene oxide starting materials since full conversion was demonstrated. The highly reactive germanium dioxide on graphene oxide support allows for the conversion of the oxide to nitride and phosphide phases. It was shown that the morphology of Ge_3_N_4_ and GeP crystalline phases depended on the loading of germanium dioxide in the GeO_2_-rGO composite. For low loadings of 20 wt.% germanium compound, only a very thin film of active material was obtained, whereas at higher loading, coatings with bimodal size distribution were demonstrated.

The homogeneous distribution of germanium compounds (exclusively) over the surface of the graphene oxide was demonstrated in all the electrode coatings. However, in the germanium phosphide and nitride electrodes, the particle size distribution was bimodal, comprised of approximately 2 nm thick homogenous coverage of the rGO surface and larger nanoparticles of around 200 nm particles densely decorating the surface. The ratio between these two phases was a function of the germanium oxide loading; only thin-film coating was formed from 20 wt.% GeO_2_ loading, while the fraction of the large particles was considerably higher for the materials prepared from the 80 wt.% GeO_2_ loading. The charge capacity was accordingly higher for the high-loading materials, but alloying pulverization was much lower for the active materials with smaller particles. Excellent stability of Ge_3_N_4_-rGO-20 and GeP-rGO-20 composites was demonstrated by 150 cycles at 100 mA g^−1^ with charge capacity loss of less than 17% and 23%, respectively, in the last 150 cycles. The higher stability of the low Ge-loaded anodes was attributed to the superior adhesion of the uniform nanoparticle coverage of the rGO and its larger specific contact area rather than to the higher carbon content, since GeO_2_ electrodes did not exhibit superior stability at low germanium loading.

Germanium is an expensive resource, and therefore, even for niche applications, its theoretical charge capacity should be approached. The Ge_3_N_4_-rGO-20 and GeP-rGO-20 demonstrate that even at a 100 mA g^−1^ rate, the conversion reaction was close to quantitative, and the almost theoretical charge capacity of the germanium compounds was approached: 2595 vs. 2465 mAh g^−1^ for Ge_3_N_4_ and 1790 vs. 1850 mAh g^−1^ for GeP. The germanium oxide was less efficiently exploited due to its lower conversion reversibility compared to GeP and Ge_3_N_4_ electrodes, following previous reports.

## Figures and Tables

**Table 1:** Elemental analysis of GeO_2_-rGO, Ge_3_N_4_-rGO, and GeP-rGO materials.

Material	C, wt.% (by CHN)	N, wt.% (by CHN)	Ge:P, at:at (by EDX)
GeO_2_-rGO-80	16.7		
GeO_2_-rGO-50	42.9		
GeO_2_-rGO-20	75.5		
Ge_3_N_4_-rGO-80	25.0	12.0	
Ge_3_N_4_-rGO-50	48.0	9.4	
Ge_3_N_4_-rGO-20	76.2	6.8	
GeP-rGO-80	13.5		1.1
GeP-rGO-50	45.1		0.9
GeP-rGO-20	74.3		1.2

## Data Availability

The data presented in this study are available in the article and Supplementary Materials.

## Supplementary Materials

The following supporting information can be downloaded at https://www.mdpi.com/article/10.3390/ijms24076860/s1.
