# Supplementary material for: Electrochemical Behavior of Reduced Graphene Oxide Supported Germanium Oxide, Germanium Nitride, and Germanium Phosphide as Lithium-Ion Battery Anodes Obtained from Highly Soluble Germanium Oxide
Source: Int J Mol Sci. 2023 Apr 6;24(7):6860. doi: 10.3390/ijms24076860 (PMC10095334; doi:10.3390/ijms24076860)
Supplement: Supplementary file 1 [file ijms-24-06860-s001.zip › ijms-2310507-supplementary.pdf]

Electrochemical Behavior of Reduced Graphene Oxide Supported Germanium Oxide, Germanium Nitride, and Germanium Phosphide as Lithium-Ion Battery Anodes Obtained from Highly Soluble Germanium Oxide

Alexey A. Mikhaylov [1], Alexander G. Medvedev [1], Dmitry A. Grishanov [2], Timur M. Fazliev [1], Vasilii Chernyshev [1], Elena A. Mel'nik [1], Tatiana A. Tripol'skaya [1], Ovadia Lev [2,3] and Petr V. Prikhodchenko [1,*]

[1] Kurnakov Institute of General and Inorganic Chemistry, Russian Academy of Sciences, Leninskii prosp. 31, 119991 Moscow, Russia
[2] The Casali Center of Applied Chemistry, The Institute of Chemistry, The Hebrew University of Jerusalem, Jerusalem 9190401, Israel
[3] The Harvey M. Krueger Family Center for Nanoscience and Nanotechnology, The Hebrew University of Jerusalem, Edmond J. Safra Campus, Jerusalem 9190401, Israel
* Correspondence: prikhman@gmail.com

Material characterization

Elemental analysis. The composition of the active electrode material was determined by complementing analytical techniques. C, H, N contents were determined by using a Perkin-Elmer 2400 series II Analyzer.

Scanning Electron Microscopy, SEM imaging was performed using the Carl Zeiss NVision 40 High-Resolution Scanning Electron Microscope (Germany) and TESCAN AMBER GMH (Czech Republic). The specimen was prepared by depositing small amount of the powder on the conductive carbon tape.

X-ray powder diffraction. XRD measurements were performed on a D8 Advance Diffractometer (Bruker AXS, Karlsruhe, Germany). The powder samples were carefully filled into low background quartz sample holders. The specimen weight was approximately 0.5 g. XRD patterns from 5° to 75° 2θ were recorded at room temperature using CuKα radiation (1.5418 Å) under the following measurement conditions: tube voltage of 40 kV, tube current of 40 mA, step scan mode with a step size 0.02° 2θ and counting time of 1 s/step. XRD patterns were processed using Diffrac Plus software.

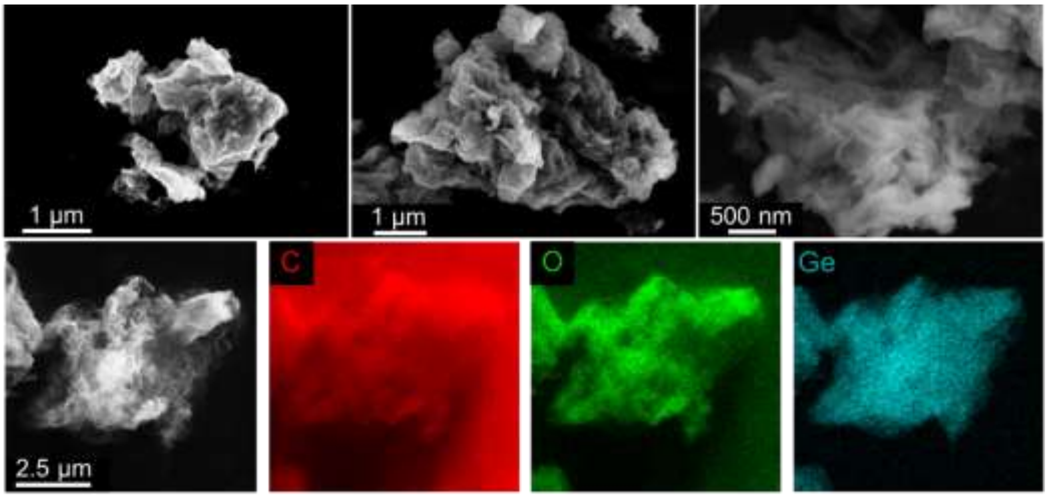

Figure S1. SEM of GeO_2-GO-80 -upper row. Energy dispersive X-ray (EDX) mapping analysis of GeO_2-rGO-80 composite lower row.

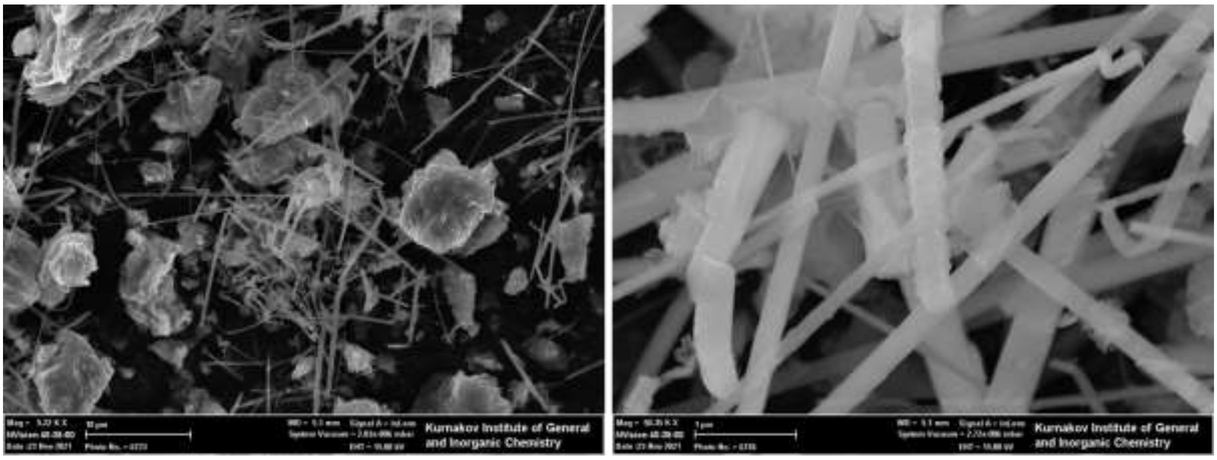

Figure S2. SEM of Ge_3N_4-rGO obtained by treatment at 700°C for 2 h.

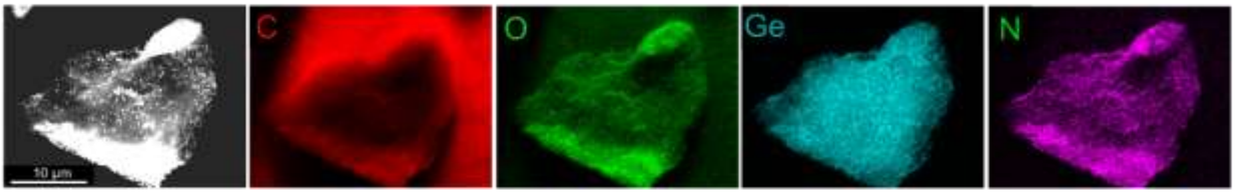

Figure S3. Energy dispersive X-ray (EDX) mapping analysis of Ge_3N_4-rGO-80 composite.

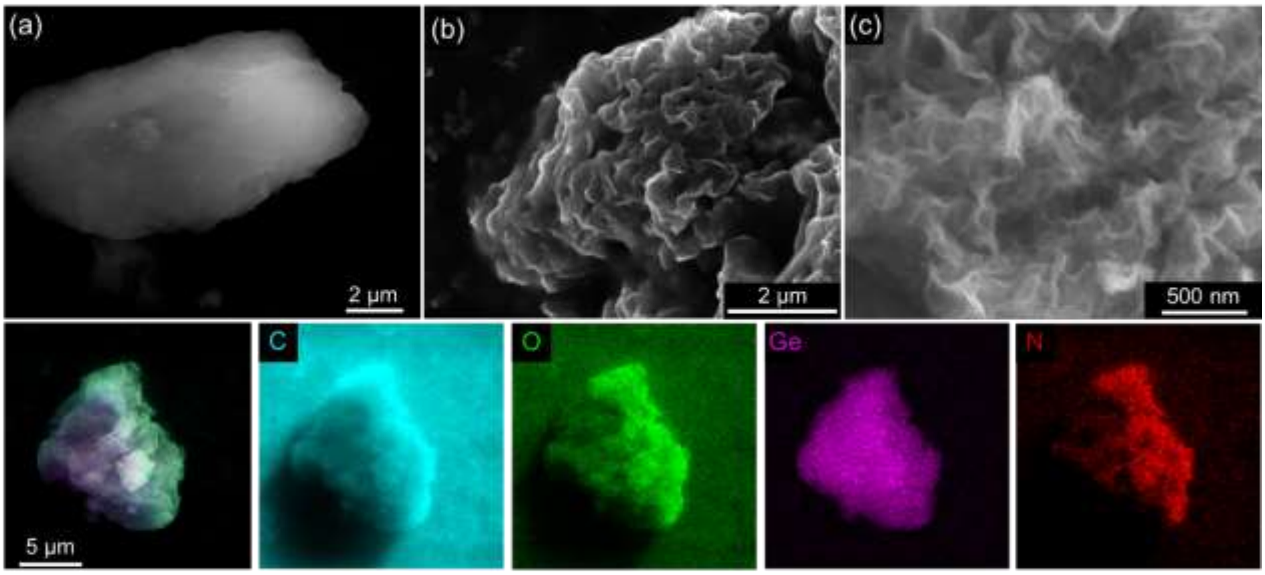

Figure S4. SEM images of Ge_3N_4-rGO-20 and energy dispersive X-ray (EDX) mapping analysis of composite.

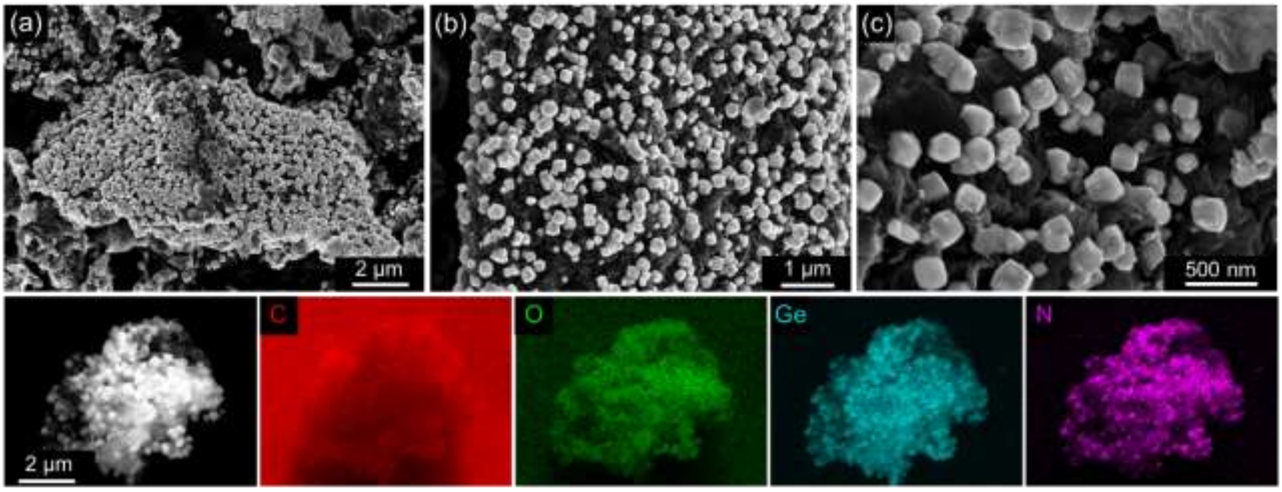

Figure S5. SEM images of Ge_3N_4-rGO-50 and energy dispersive X-ray (EDX) mapping.

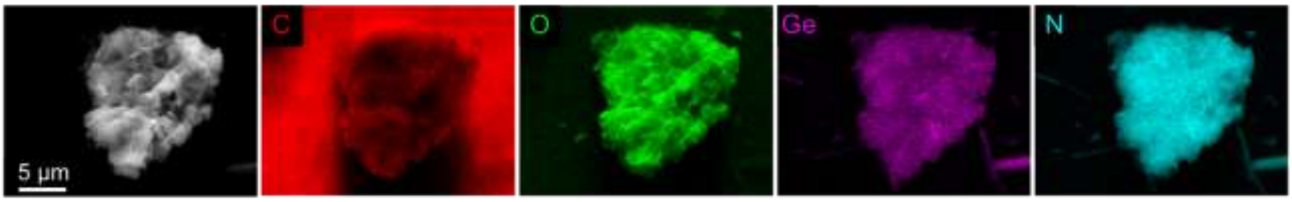

Figure S6. Energy dispersive X-ray (EDX) mapping analysis of GeP-rGO-80 composite.

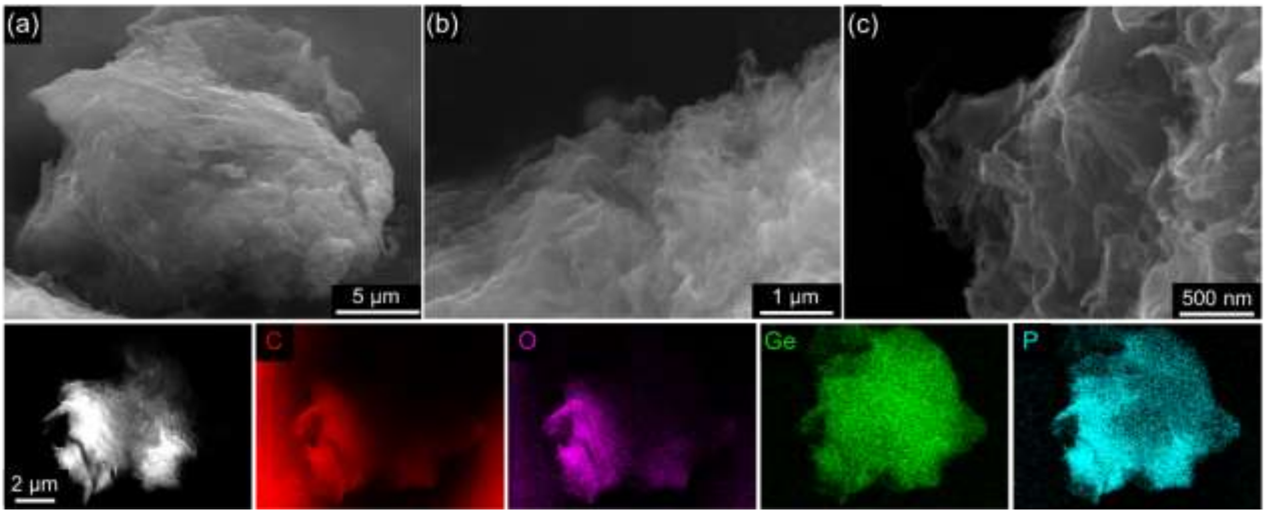

Figure S7. SEM images of GeP-rGO-20 and energy dispersive X-ray (EDX) mapping analysis of composite.

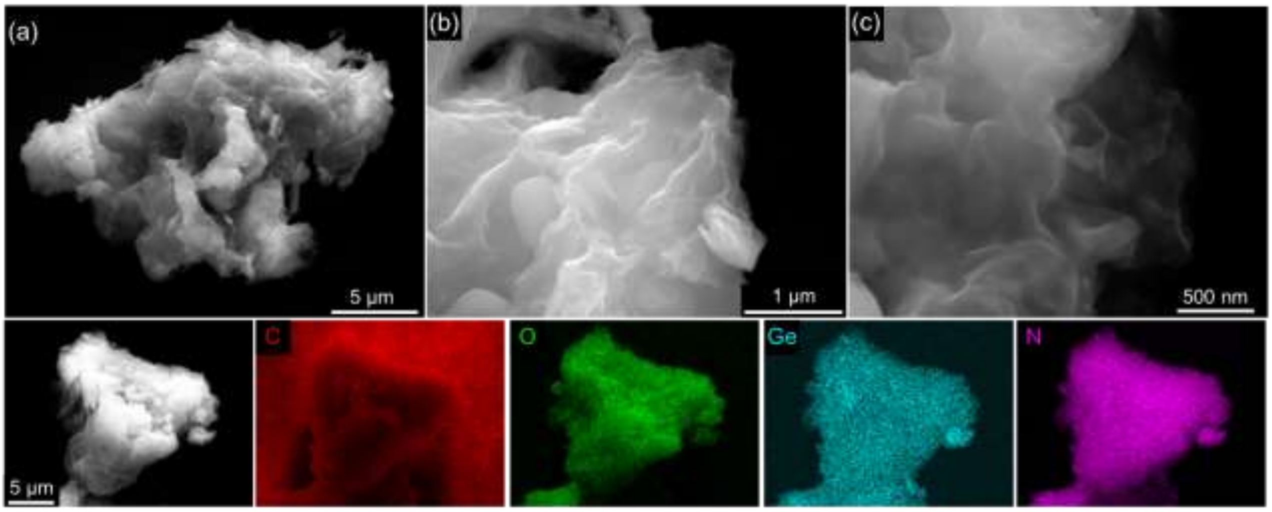

Figure S8. SEM images of GeP-rGO-50 and energy dispersive X-ray (EDX) mapping analysis of composite.

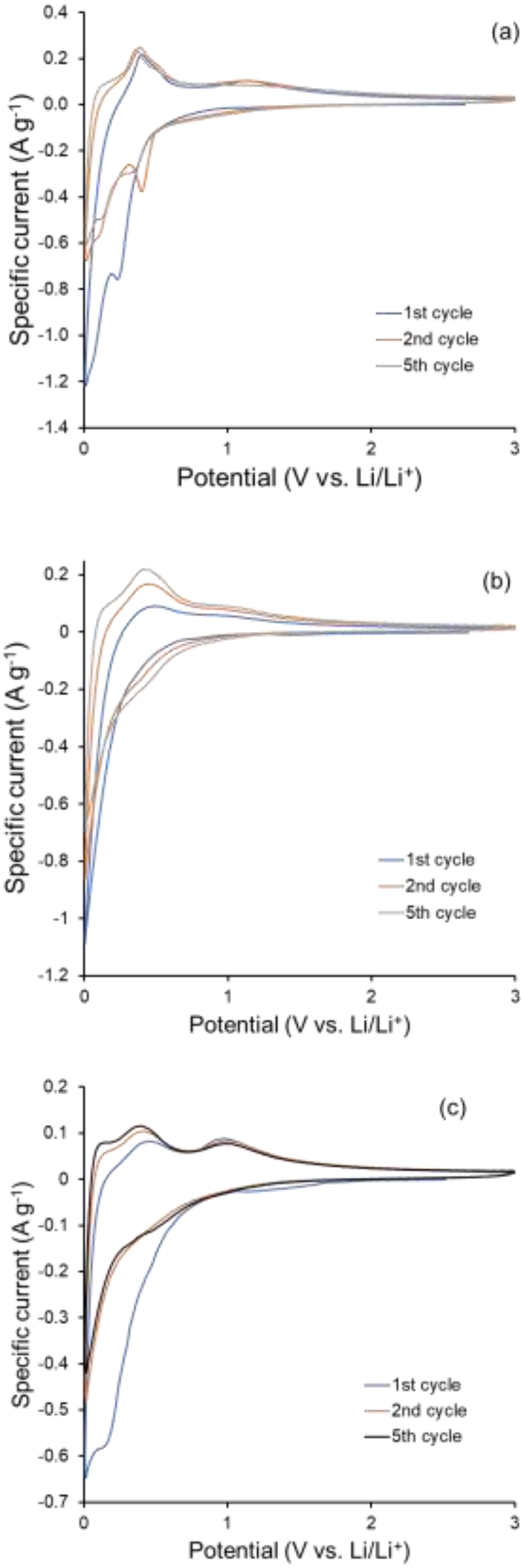

Figure S9. CV curves of GeO_2-rGO-20 (a); Ge_3N_4-rGO-20 (b) and GeP-rGO-20 (c).

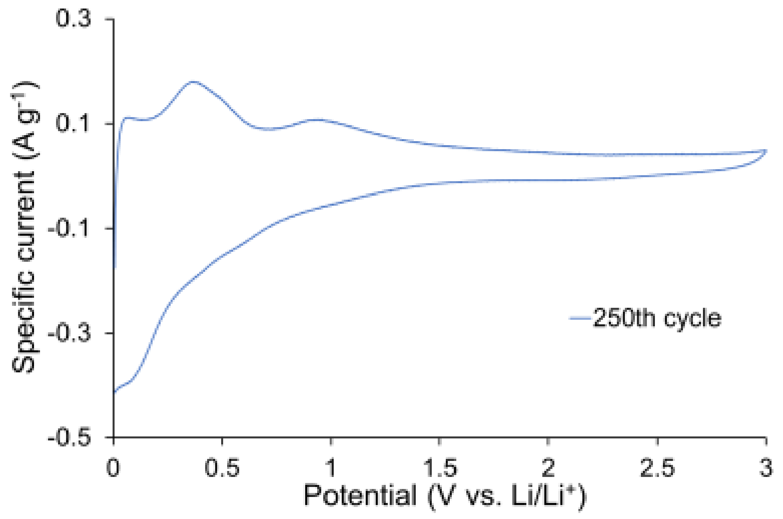

Figure S10. CV curve of Ge_3N_4-rGO-80 after 250 discharge-charge cycles at 100 mA g^{-1} with scan rate 0.1 mV s^{-1} in the 0.005-3.0 V potential range vs. Li/Li^+.

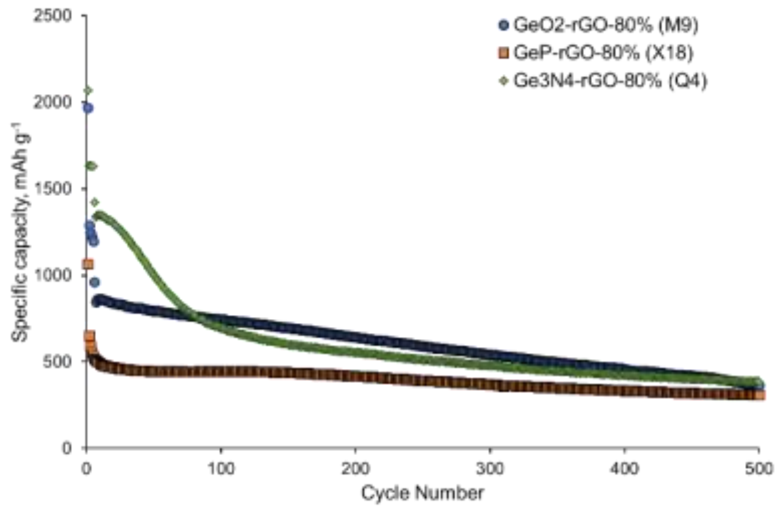

Figure S11. Electrode performance of GeO_2-rGO-80, Ge_3N_4-rGO-80 and GeP-rGO-80 at rate of 1000 mA g^{-1} between 0 and 3 V vs Li/Li^+.

Average voltage hysteresis

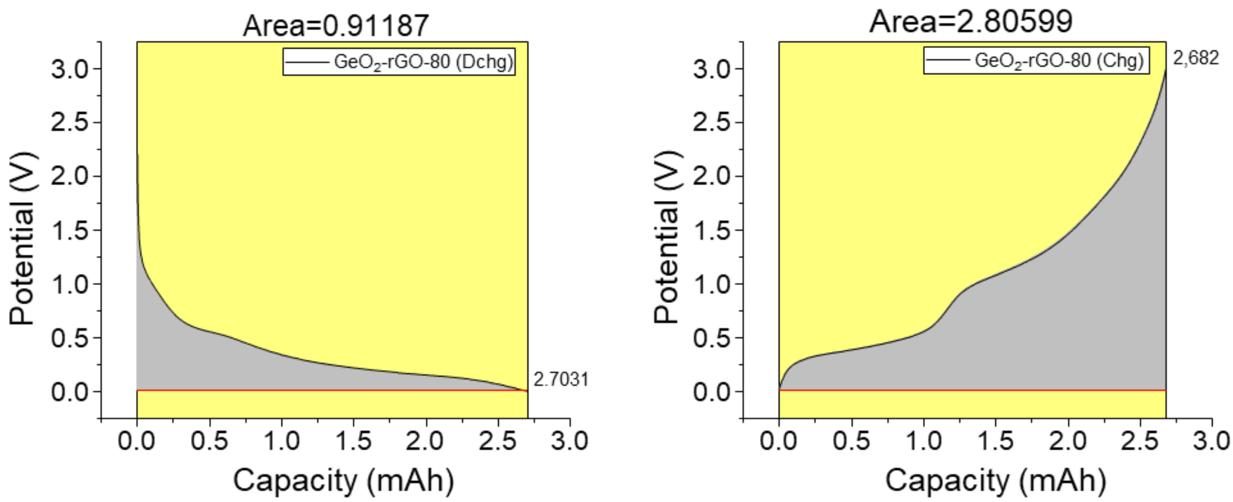

Figure S12. 35th discharge and charge curves of GeO_2-rGO-80.

The average voltage hysteresis can be readily calculated from the galvanostatic curves of Figures 3d-f and S12.

$$\overline{\Delta V} = \frac{\int_{charging} V dq}{Q_{charging}} - \frac{\int_{discharging} V dq}{Q_{discharging}} \quad \text{(S1)}$$

The first and second terms on the right-hand side of Equation S1 correspond to the average charging and discharging voltage. q is the specific charge (Coul) and the integral Q = $\int dq$ is the overall specific charge capacity. $\overline{\Delta V}$ was calculated at the 30th cycle (the anodes were cycled between 0.005 and 3.0 V at 100 mA g^{-1}).
